# Distinct Gut Microbiota Induced by Different Fat-to-Sugar-Ratio High-Energy Diets Share Similar Pro-obesity Genetic and Metabolite Profiles in Prediabetic Mice

**DOI:** 10.1128/mSystems.00219-19

**Published:** 2019-10-08

**Authors:** Kai Shan, Hongyan Qu, Keru Zhou, Liangfang Wang, Congmin Zhu, Haiqin Chen, Zhennan Gu, Jing Cui, Guoling Fu, Jiaqi Li, Heyan Chen, Rong Wang, Yumin Qi, Wei Chen, Yong Q. Chen

**Affiliations:** a Wuxi School of Medicine, Jiangnan University, Wuxi, Jiangsu Province, China; b School of Food Science and Technology, Jiangnan University, Wuxi, Jiangsu Province, China; c School of Science, Jiangnan University, Wuxi, Jiangsu Province, China; d MOE Key Laboratory of Bioinformatics and Bioinformatics Division, Center for Synthetic & Systems Biology, TNLIST/Department of Automation, Tsinghua University, Beijing, China; e Beijing Innovation Centre of Food Nutrition and Human Health, Beijing Technology and Business University, Beijing, China; University of California San Diego

**Keywords:** high-energy diet, prediabetes, gut microbiota, metagenome, metabolome

## Abstract

Gut microbiota play important roles in host metabolism, especially in diabetes. However, why different diets lead to similar diabetic states despite being associated with different microbiota is not clear. Mice were fed two high-energy diets (HED) with the same energy density but different fat-to-sugar ratios to determine the associations between the microbiota and early-stage metabolic syndrome. The two diets resulted in different microbiota but similar diabetic states. Interestingly, the microbial gene profiles were not significantly different, and many common metabolites were identified, including l-aspartic acid, cholestan-3-ol (5β, 3α), and campesterol, which have been associated with lipogenesis and inflammation. Our study suggests that different metabolic-syndrome-inducing diets may result in different microbiota but similar microbiomes and metabolomes. This suggests that the metagenome and metabolome are crucial for the prognosis and pathogenesis of obesity and metabolic syndrome.

**IMPORTANCE** Various types of diet can lead to type 2 diabetes. The gut microbiota in type 2 diabetic patients are also different. So, two questions arise: whether there are any commonalities between gut microbiota induced by different pro-obese diets and whether these commonalities lead to disease. Here we found that high-energy diets with two different fat-to-sugar ratios can both cause obesity and prediabetes but enrich different gut microbiota. Still, these different gut microbiota have similar genetic and metabolite compositions. The microbial metabolites in common between the diets modulate lipid accumulation and macrophage inflammation *in vivo* and *in vitro*. This work suggests that studies that only use 16S rRNA amplicon sequencing to determine how the microbes respond to diet and associate with diabetic state are missing vital information.

## Abstract (precis)

Various types of diet can lead to type 2 diabetes. The gut microbiota in type 2 diabetic patients are also different. So, two questions arise: whether there are any commonalities between gut microbiota induced by different pro-obese diets and whether these commonalities lead to disease. Here we found that high-energy diets with two different fat-to-sugar ratios can both cause obesity and prediabetes but enrich different gut microbiota. Still, these different gut microbiota have similar genetic and metabolite compositions. The microbial metabolites in common between the diets modulate lipid accumulation and macrophage inflammation *in vivo* and *in vitro*. This work suggests that studies that only use 16S rRNA amplicon sequencing to determine how the microbes respond to diet and associate with diabetic state are missing vital information.

## INTRODUCTION

Obesity has become a pandemic (1). It is well known that a sedentary lifestyle and especially a high-energy diet are important pro-obesity factors. Evidence suggests that gut microbiota play important roles in metabolism, whereas different diets shape different gut microbiomes (2). Therefore, the following question arises: why can diverse foods and cooking methods around the globe result in the same phenotype, namely, obesity?

Excess energy intake from a high-fat diet (HFD) can be naturally considered the cause of metabolic syndrome, but further studies indicate that the dietary fat, not energy, is the critical factor in HFD-induced experimental obesity (3–6). It has been well established that dietary fat can regulate genes related to lipid metabolism and hormone signaling related to appetite via various molecular pathways (6–9). Lipid sensor and related pathway research has been especially focused on the cross talk among peroxisome proliferator-activated receptors (PPARs), sterol regulatory element binding proteins (SREBPs), and the AMP-activated protein kinase (AMPK) pathway (10–12). In addition to regulating fat storage, these pathways modulate macrophage-associated chronic inflammation and thus mediate insulin resistance (13–15). Beyond the influence on host, responses of gut microorganisms to dietary fat are also reported (16). In addition to fat, excessive sugar in Western diets also shows a positive relationship to the risk of metabolic diseases. However, the mechanism is still unclear (17–20).

The human intestine hosts tens of trillions of microorganisms which contribute over 100 times more genes than does the human genome (21). Changes in gut microbial diversity and composition are associated with several diseases, including obesity and diabetes, according to clinical and basic research (22). Pro-obesity microbiota promote energy harvest and storage via influencing bile acid transformation, fermenting, and providing vitamins and amino acids for the host (23). But the definitions of metabolic-syndrome-associated microbiota are contradictory because of the differences between hosts, diversities of metabolic diseases, and dynamics of gut microbiota in different pathological stages. For example, *Lactobacillus* is significantly increased in Chinese patients with type 2 diabetes (T2D) but is normal in T2D patients from Denmark or Sweden (24). Thus, to obtain clinically significant information regarding gut microbiota, we prefer to focus on the functional change of microbiota at the critical stage of pathogenesis. Prediabetes, which is generally defined as impaired fasting blood glucose and/or impaired glucose tolerance and/or abnormal HbA1c, is a critical control point in both pathogenesis and prevention of T2D (25), and microbes at this time point deserve more attention.

HFD-fed rodents with various genotypes are widely used animal models for studying metabolic syndrome (8, 26, 27). Mice fed an HFD show weight gain, metabolic efficiency reduction, hyperglycemia, and insulin resistance which simulates well the pathogenesis of human obesity and T2D (26, 27). However, recent research showed that this diet-induced obesity (DIO) in mice (especially the widely used C57BL6/J mice) could not simulate T2D in humans due to the absent of islet atrophy (28, 29). Thus, to avoid this limitation, mice with at least one normal index among fasting blood glucose, glucose tolerance, and HbA1c can be regarded as a credible model of prediabetes. In this study, we used a high-fat diet and a high-sucrose diet, which were equal-energy, high-energy diets, to explore their effects on gut microbiota and metagenomics at the prediabetes stage. We found that although these two diets shaped distinct gut microbiota, they induced similar metagenomic expression and metabolite profiles. We showed that some common metabolites could modulate lipid accumulation and macrophage activation.

## RESULTS

### The HF diet and HS diet both induce prediabetes in mice.

**FIG 1:**
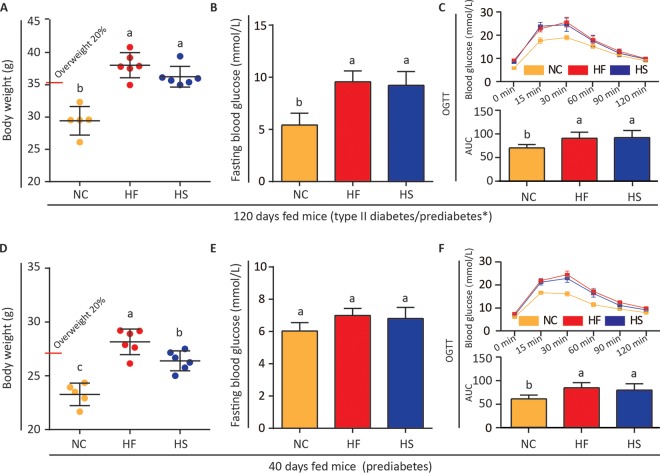
The HF diet and HS diet both induced prediabetes in mice. (A and D) Body weights of mice fed different diets for 120 days and 40 days. (B and E) Fasting blood glucose values of mice on the 120th day and 40th day. (C and F) Oral glucose tolerance test of mice fed different diets for 120 days and 40 days. *, the phenotype of C57BL6/J mice with long-term HFD is controversial. Some researchers regarded it as a type II diabetes model (26), while others considered it prediabetes (28, 29). One-way ANOVA was adopted to compare the difference of these groups. Groups marked with different letters are significantly different (*P* < 0.05).

Gut microbiota in mice with diet-induced obesity have been studied extensively. Microbial composition and functions in prediabetic mice are less clear. Prediabetes is signified by impaired fasting blood glucose, impaired glucose tolerance, or abnormal HbA1c (25). After 120 days of regular high-energy diet (HED) feeding, mice exhibited both overweight and blood glucose disorder (Fig. 1A to C), features of obesity and metabolic syndrome (30, 31). At 40 days, mice fed an HF but not HS diet showed overweight (Fig. 1D). However, signs of alteration in fasting blood glucose and impaired glucose tolerance had appeared (Fig. 1E and F). These results suggest that HF and HS diets may induce metabolic disorders with different time courses. We consider 40-day fed mice as having prediabetes and thus focus our subsequent experiments at this stage.

### Different fat-to-sugar ratios shape distinct gut microbiota in prediabetic mice.

**FIG 2:**
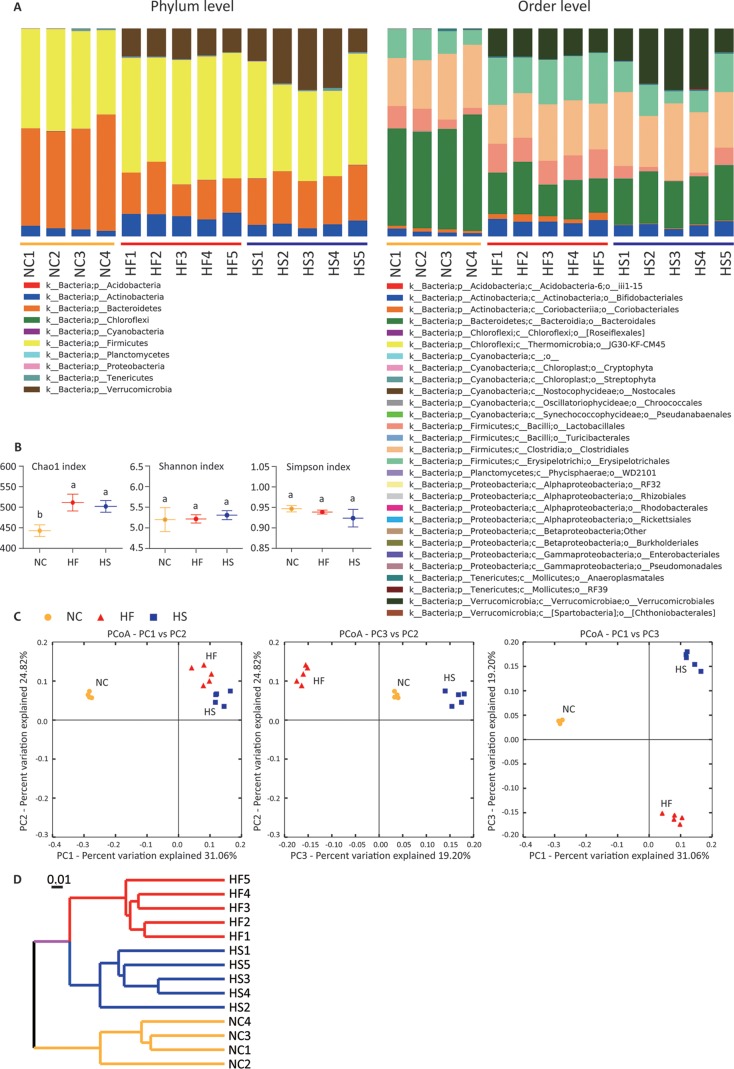

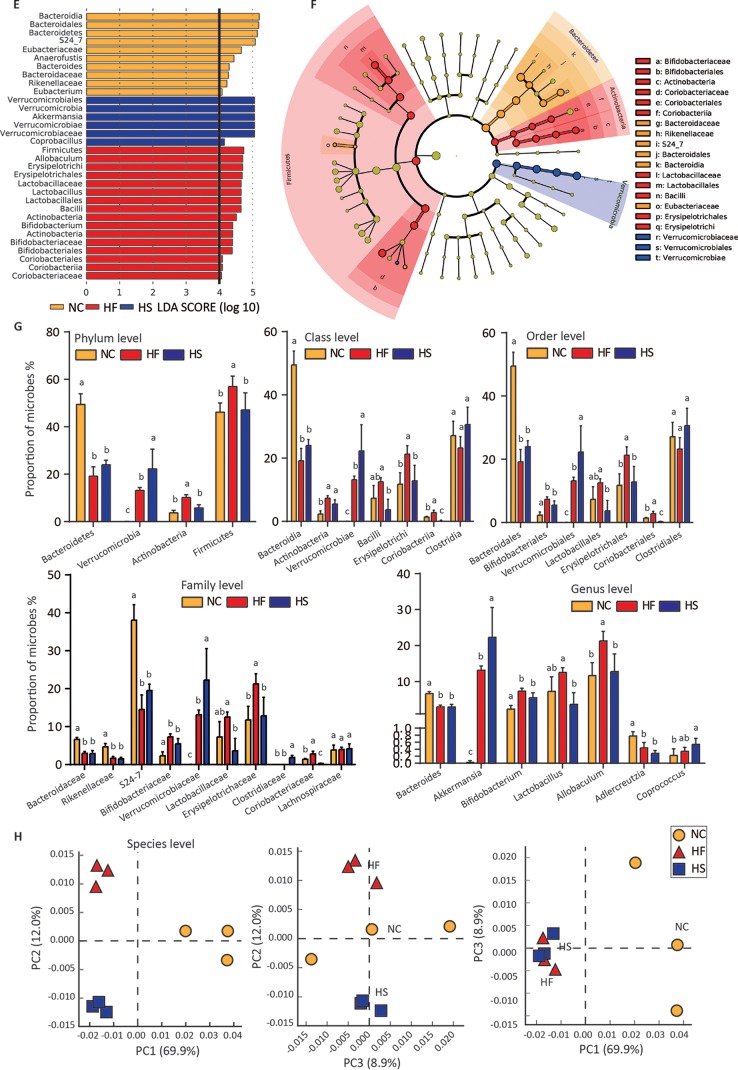
Different fat-to-sugar ratios shape distinct gut microbiota in prediabetic mice. (A) Phylum- and order-level taxonomic distributions of fecal microbiota from mice fed different diets for 40 days. Stacked columns show the mean of abundance of a given genus as a percentage of the total bacterial sequences in the corresponding group. (B) Analysis of alpha-diversity in mice fed NC, HF, and HS diets diversity by Chao1, Shannon, and Simpson estimators. Groups with different letters have significant differences (one-way ANOVA with Bonferroni test, *P < *0.05). (C) Principal-coordinate analysis (PCoA) of samples based on relative abundance of OTUs using weighted UniFrac metric (*R*^2^ = 0.84756). (D) Weighted UniFrac tree analysis of samples. The hierarchical clustering structure helps to determine the similarity of the microbial communities between different groups. (E) Linear discriminative analysis (LDA) effect size (LefSe) analysis among the NC (orange), HF (red), and HS (blue) diets. (F) Cardiogram showing differentially abundant taxonomic clades with an LDA score of >4.0 among cases with a *P* value of *<*0.05. (G) Relative abundances of bacterial taxa at the different levels. Groups with different letters have significant differences (one-way ANOVA with Tukey test, *P < *0.05). (H) Metagenomics revealed microbial composition at the species level. Shown is principal-component analysis of the matrix data for species analysis from mice fed different diets. Accordingly, mice that received the same diet can be obviously clustered. In bar charts, data are shown as means ± SDs.

Given that HF and HS feeding show some similar metabolic alterations in the host, we wondered whether these diets similarly reshape gut microbiota as well. Fecal samples were collected and used for 16S rRNA gene sequencing. Interestingly, we found distinct changes between mice fed HF and HS diets at the phylum and order levels (Fig. 2A). The Chao1 index increased significantly in the HF and HS groups, indicating an upregulation of microflora richness (Fig. 2B). Although the Shannon and Simpson indices suggested no marked difference in gut microbiota diversity between mice fed normal control chow (NC) and HEDs (Fig. 2B), samples were clearly clustered according to their diet (Fig. 2C). Weighted UniFrac tree analysis supported the clustering and showed that the control diet cluster was far away from the two HED clusters (Fig. 2D). These results indicate that diets with different fat-to-sugar ratios shape distinct gut microbiota in prediabetic mice.

To further study the changes of microbiota, we used the linear discriminant analysis effect size (LEfSe) tool to characterize the differences of gut microbiota among mice fed NC, HF, and HS diets (Fig. 2E and F). In general, there were four significantly different phyla, of which *Bacteroidetes* was high in the control group, *Actinobacteria* and *Firmicutes* were enriched in the HF and HS groups, respectively, and *Verrucomicrobia* was not detected in the control (Fig. 2E to G). According to the LEfSe analysis, these abundant taxa can be considered potential biomarkers (linear discriminative analysis [LDA] score > 4.0; *P* < 0.05) (Fig. 2E). *Bacteroidaceae* (*Bacteroidetes*) is a potential biomarker for the NC group. *Bifidobacteriaceae* (*Actinobacteria*) and *Lactobacillaceae* (*Firmicutes*) are representatives of the HF group, and *Verrucomicrobiaceae* (*Verrucomicrobia*) is a hallmark of the HS group (Fig. 2E and F).

Changes of microbiota at the class, order, family, and genus levels basically followed similar trends except in the case of *Adlercreutzia*, which was reduced at the genus level in the HF and HS groups, and in the cases of *Coprococcus* and *Clostridiaceae*, which were increased at the genus and family levels, respectively, in the HS group (Fig. 2G). To further understand the remodeling of gut microbiota, shotgun metagenomic sequencing was performed to observe the changes at the species level. Principal-component analysis (PCA) also revealed that microbiota are also clustered according to diet (Fig. 2H). It is also worth noting that changes at the genus level do not represent changes in all species of the genus (see Fig. S1 in the supplemental material). These results indicate that HF and HS diets induce two distinct patterns of obesity-associated gut microbiota in prediabetic mice.

10.1128/mSystems.00219-19.1 FIG S1 Heat maps indicating changes of gut microbes at the species level from mice fed NC, HFD, and HSD are shown. Twenty species of the highest abundance of each genus were processed to calculate statistical differences, and species changed with significant difference are listed (ANOVA, LSD, *P ≤ *0.05). Download FIG S1, TIF file, 1.6 MB. Copyright © 2019 Shan et al. 2019 Shan et al. This content is distributed under the terms of the Creative Commons Attribution 4.0 International license.

### HF and HS diets enrich similar gene profiles.

**FIG 3:**
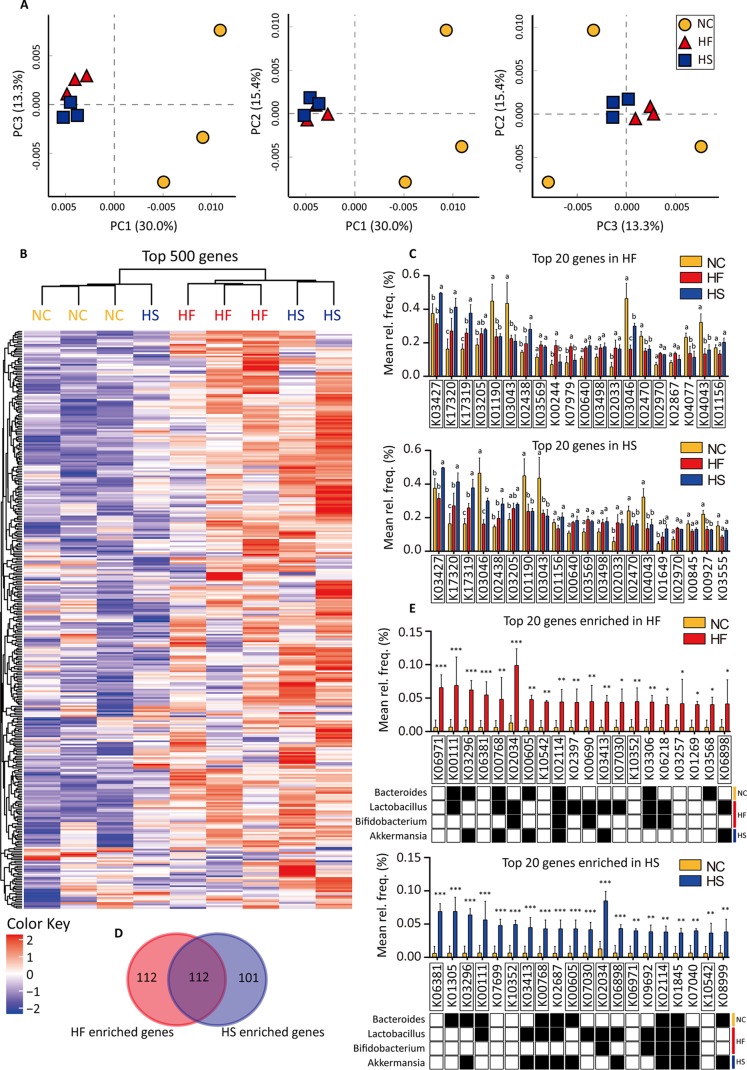

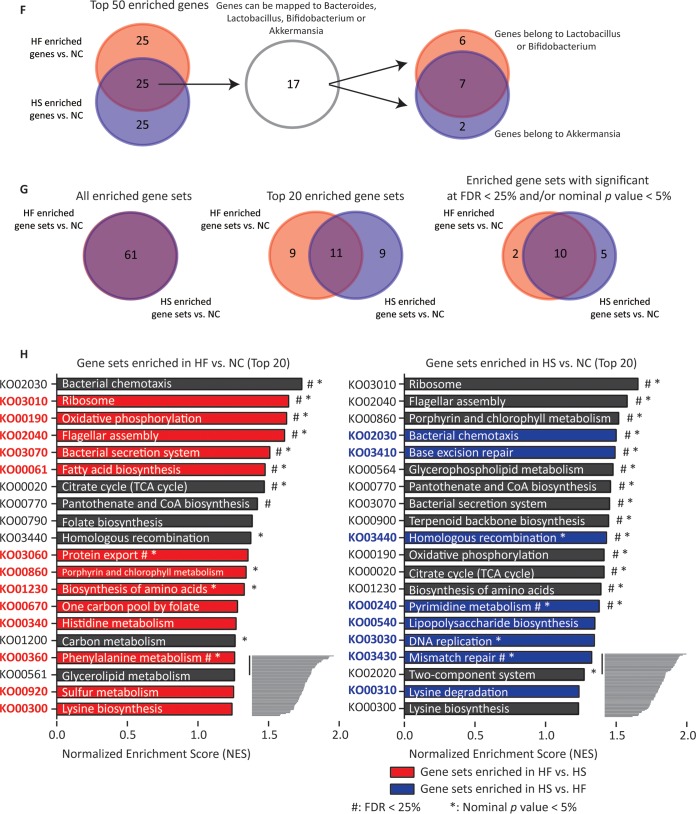
HF and HS diets enrich similar gene profiles. All genes and pathways were annotated using the KEGG database. (A) Principal-component analysis (PCA) of the matrix data for gene analysis. Gene profiles of the NC, HF, and HS groups were clustered in the PCA plotting. (B) A heat map is used to show the top 500 genes which are abundant in the NC, HF, and HS groups. ANOVA was performed to discover differences, and Benjamini FDR was used for correction for multiple tests. A *P* value of *<*0.05 was considered significant. Significantly different genes are listed. Listed genes in red are HFD-enriched genes, while listed genes in blue are HSD-enriched genes. ANOSIM statistic *R* values were calculated. (C) Twenty genes with the highest abundances in the HF and HS groups are shown. Two-way ANOVA was performed to discover differences, and significantly different groups are labeled with different letters (*P < *0.05). (D) Venn diagram showing the relationship between HFD-enriched genes (*t* test, *P < *0.05, fold > 1) and HSD-enriched genes (*t* test, *P < *0.05, fold > 1). (E) Twenty genes with the highest fold differences between the HF and NC groups and between the HS and NC groups. Two-way ANOVA was performed to discover differences, and significantly different groups are indicated. *, *P < *0.05; **, *P < *0.01; ***, *P < *0.001. (F) Venn diagrams show the genes mutually enriched in the HF and HS groups (*P < *0.05). The distributions of these genes in significant microbes of the HF and HS groups are also indicated. (G) Venn diagrams show the relationship between HFD- and HSD-enriched gene sets, top 20 enriched gene sets, and enriched gene sets with significance at an FDR of <25% and/or nominal *P* value of <5%. (H) Twenty gene sets (pathways) with the highest abundance enriched in the HF and HS groups (versus NC). Red and blue bars showed the enriched gene sets in the HF (versus HS) and HS (versus HF) groups. “*” and “#” indicate result of statistical analysis between the HF and NC groups or between the HS and NC groups. ANOVA was performed to discover differences, and Benjamini FDR was used for correction for multiple tests. #, FDR > 25%; *, *P* value < 0.05. Calculation of gene set enrichment was performed via GESA. Normalized enrichment score (NES) was used to calculate enrichment degree following the formula NES = actual enrichment score/mean (ESs against all permutations of the data set). GSEA calculates the ES by walking down the ranked list of genes, increasing a running-sum statistic when a gene is in the gene set and decreasing it when it is not. In bar charts, data are shown as means ± SDs.

To explore the functional consequences of HED feeding, we performed shotgun metagenomic sequencing of fecal samples. A total of 3,821 genes were detected in total. PCA indicated that the gene profiles of the HF and HS groups were close but were very different from that of the control, although the bacterial species were significantly different between these two groups (Fig. 2H and Fig. 3A). Expression of the 500 most abundant genes was shown as a heat map, and the analysis of similarity (ANOSIM) statistic R (Bray-Curtis distance) also indicated that gene profiles in the HF and HS groups shared more similarities [R_(NC vs. HF)_ = 1, R_(NC vs. HS)_ = 0.815, and R_(HF vs. HS)_ = 0.296] (Fig. 3B). The anosim statistic R is based on the difference of mean ranks between groups (r_B) and within groups (r_W): R = (r_B − r_W)/[N(N − 1)/4]. So R will be in the interval -1 to +1, with value 0 indicating completely random grouping. Sixteen of the 20 highest-expressed genes in the HF and HS groups overlapped; these were K03427 (*hsdM*), K17320 (*lplC*), K17319 (*lplB*), K03205 (*virD4*), K01190 (*lacZ*), K03043 (*rpoB*), K02438 (*glgX*), K03569 (*mreB*), K00640 (*cysE*), K03498 (*trkH*, *trkG*, and *ktrB*), K02033 (ABC.PE.P), K03046 (*rpoC*), K02470 (*gyrB*), K02970 (*RP-S21*, *MRPS21*, and *rpsU*), K04043 (*dnaK* and *HSPA9*), and K01156 (*res*) (Fig. 3C). Moreover, when 224 genes enriched in the HF group were compared with 213 genese enriched in the HS group, 112 genes were found to be the same (Fig. 3D and Fig. S2 to S4). The 20 most enriched genes in HF and HS groups also overlapped (Fig. 3E). These genes were then mapped to the Kyoto Encyclopedia of Genes and Genomes (KEGG) annotation database. We noticed that most genes enriched in the HF group could be found in *Lactobacillus* or *Bifidobacterium*, while most genes enriched in the HS group were from *Akkermansia* (Fig. 3E), and four genes (K00768, K02114, K03413 and K06898) were in both the HF and HS groups (Fig. 3E). When the top 50 enriched genes were considered, 25 genes were found in both the HF and HS groups, of which 17 genes could be mapped to *Lactobacillus*, *Bifidobacterium*, and *Akkermansia* (Fig. 3F).

10.1128/mSystems.00219-19.2 FIG S2 Abundances of KEGG-annotated genes from HFD- and NC-fed mice are compared. Genes changed with significant difference are listed (*P ≤ *0.05). Download FIG S2, TIF file, 2.4 MB. Copyright © 2019 Shan et al. 2019 Shan et al. This content is distributed under the terms of the Creative Commons Attribution 4.0 International license.

Next we grouped all genes into pathway sets and found that 61 gene sets were in common between the sets enriched in the HF and HS groups. Narrowing down to 20 gene sets with the highest normalized enrichment score (NES) or significant sets according to false-discovery rate (FDR) (<25%) and/or *P* value (5%), the similarity seemed even greater (Fig. 3G). Common pathways were mainly involved in energy metabolism and biosynthesis of lipid and amino acids (Fig. 3H and Fig. S5). These results indicate that HF and HS diets enrich similar gene profiles despite shaping different microbiota.

10.1128/mSystems.00219-19.3 FIG S3 Abundances of KEGG-annotated genes from HSD- and NC-fed mice are compared. Genes changed with significant difference are listed (*P ≤ *0.05). Download FIG S3, TIF file, 2.5 MB. Copyright © 2019 Shan et al. 2019 Shan et al. This content is distributed under the terms of the Creative Commons Attribution 4.0 International license.

10.1128/mSystems.00219-19.4 FIG S4 Abundances of KEGG-annotated genes from HFD- and HS-fed mice are compared. Genes changed with significant difference are listed (*P ≤ *0.05). Download FIG S4, TIF file, 2.9 MB. Copyright © 2019 Shan et al. 2019 Shan et al. This content is distributed under the terms of the Creative Commons Attribution 4.0 International license.

10.1128/mSystems.00219-19.5 FIG S5 HED-enriched genes and related metabolites were mapped in pathway networks (KEGG resource). Red, HFD-enriched genes and pathways; pink, HSD-enriched genes and pathways. Colorful squares indicate related metabolites. Download FIG S5, TIF file, 2.1 MB. Copyright © 2019 Shan et al. 2019 Shan et al. This content is distributed under the terms of the Creative Commons Attribution 4.0 International license.

Although HF and HS diet-enriched genes sets were very similar, some differences exist. Comparison between the HF and HS groups also revealed some significantly different gene sets which are shown as red bars (enriched in the HF group) or blue bars (enriched in the HS group) in Fig. 3H. The HFD preferentially enriched genes involved in energy metabolism and amino acid metabolism, while the HSD tended to increase nucleotide metabolism-related genes (Fig. 3H and Fig. S6).

10.1128/mSystems.00219-19.6 FIG S6 HED-enriched genes and related metabolites were merged in pathway networks (KEGG resource). Red, HFD-enriched genes and pathways; pink, HSD-enriched genes and pathways. Colorful squares indicate related metabolites. Download FIG S6, PDF file, 1.5 MB. Copyright © 2019 Shan et al. 2019 Shan et al. This content is distributed under the terms of the Creative Commons Attribution 4.0 International license.

### HF and HS induce similar metabolite profiles.

**FIG 4:**
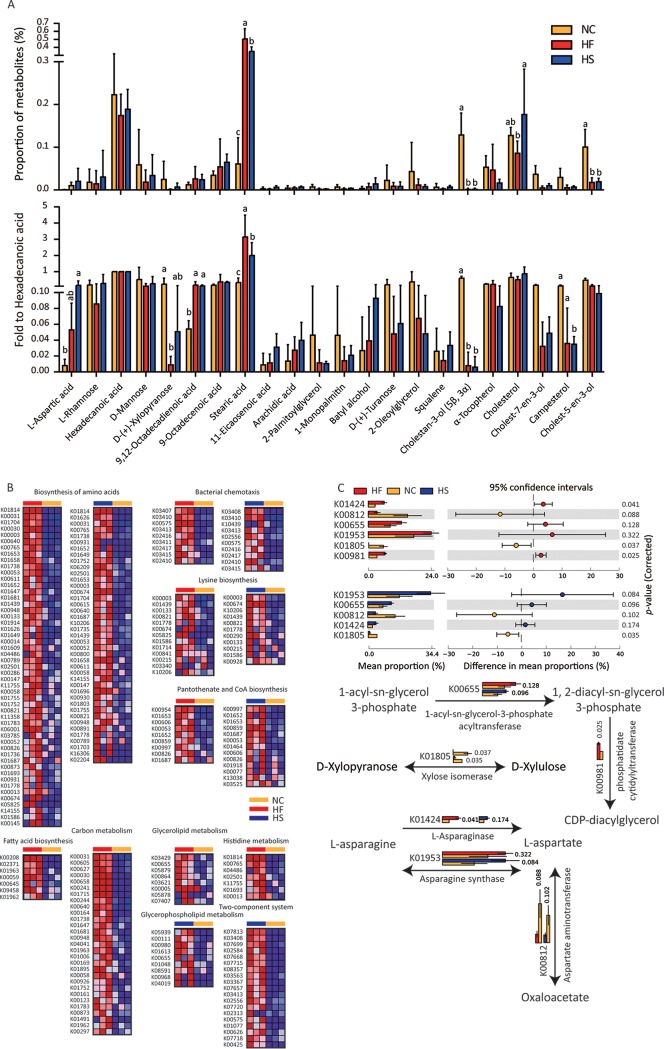
HF and HS model similar metabolite profiles. (A) GC-MS was used to analyze weak polar fecal metabolites. (Top) Area normalized metabolites; (bottom) hexadecanoic acid normalized metabolites. Groups with different letters have significant differences (one-way ANOVA with Bonferroni test, *P < *0.05). (B) To show the changes of genes in metabolite-associated pathways, a heat map is used to show the significantly different genes enriched by the HFD and/or HSD (versus NC). ANOVA was performed to discover differences, and Benjamini FDR was used for correction for multiple tests. *P < *0.05 was considered significant. (C) Genes and pathways involved in glycerolipid, d-xylopyranose, and l-aspartic acid metabolism were shown. Bar charts show the comparison of KEGG gene abundance using metagenomic sequencing data. In the bar chart, data are shown as means ± SDs.

Polar fecal metabolites such as short-chain fatty acids have been studied extensively in obese patients. Thus, we mainly focused on weak polar fecal metabolites in the present study. We found that proportion of stearic acid was increased while d-(+)-xylopyranose (d-xylose), cholestan-3-ol (5β, 3α), and cholest-5-en-3-ol were decreased in HED-fed mice (Fig. 4A). When normalized with hexadecanoic acid (one of the main components in lard), the increase of 9,12-octadecadienoic acid and stearic acid and reduction of cholestan-3-ol (5β, 3α) were significant. Furthermore, l-aspartic acid, cholest-7-en-3-ol, and campesterol seemed to be changed in HF and HS group mouse feces, although the differences were not significant in both groups (Fig. 4A). In general, metabolites induced by HF and HS diets changed, with similar trends. Increases in amino acids as well as fatty acid-related compounds and decreases in cholesterol derivatives were the principal characteristics.

Enrichment of KO02030 (bacterial chemotaxis), KO00770 (pantothenate and coenzyme A [CoA] biosynthesis), KO01230 (biosynthesis of amino acids), KO00340 (histidine metabolism), KO01200 (carbon metabolism), KO00300 (lysine biosynthesis) and KO02020 (two-component system) seemed to be consistent with the increase of l-aspartic acid. Enrichment of KO00061 (fatty acid biosynthesis), KO00561 (glycerolipid metabolism), and KO00564 (glycerophospholipid metabolism) may correspond to the increase of 9,12-octadecadienoic acid and stearic acid (Fig. 3H, Fig. 4B, and Fig. S5 and S6).

Changes of genes and pathways were closely related to metabolites. 1-Acyl-*sn*-glycerol-3-phosphate acyltransferase (K00655) and phosphatidate cytidylyltransferase (K00981), which were reduced in HED groups, might contribute to the changes of various fatty acids and related lipids (Fig. 3H and Fig. 4B and C). Changes of l-asparaginase (K01424), asparagine synthase (K01953) and aspartate aminotransferase (K00812) might be responsible for the increase of l-aspartic acid (Fig. 3H and Fig. 4B and C). Downregulation of xylose isomerase (K01805), which converts d-xylopyranose to d-xylulose, might result in the reduction of d-xylopyranose (Fig. 3H and Fig. 4B and C). Furthermore, l-aspartic acid can be the substrate of various secondary metabolic pathways (KO01230, KO00240, KO02030, KO00300, KO00770, and KO01200) which were also enriched (Fig. 3H and Fig. S5 and S6).

### HED-modulated microbes and metabolites influence lipid accumulation.

**FIG 5:**
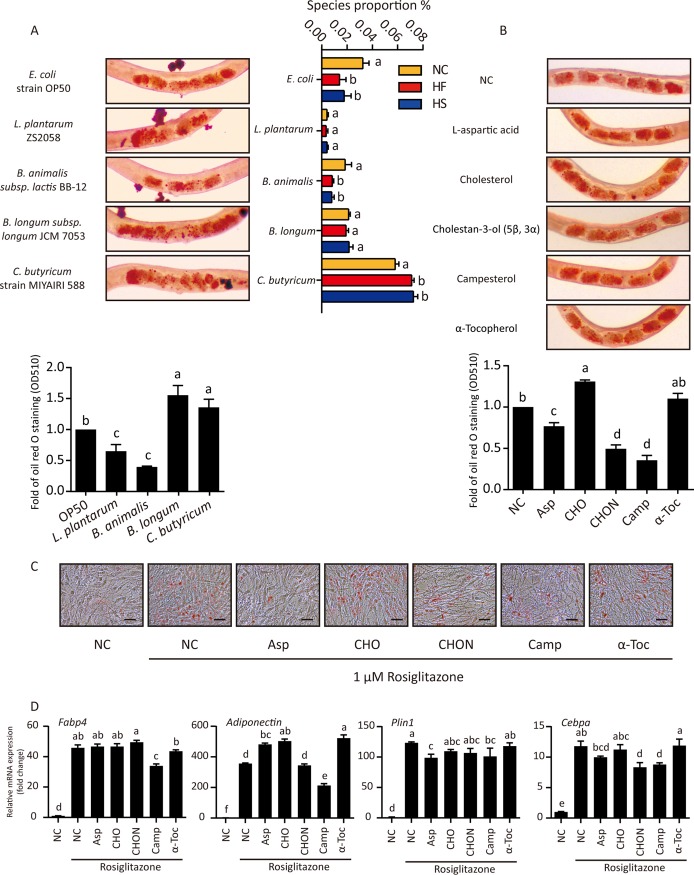
HED-modulated microbes and metabolites influence lipid accumulation. (A) (Left) Synchronized nematodes were cultured with different bacteria for 3 days and then stained with oil red O. Typical nematodes are shown in micrographs. After washing, oil red O from 50 nematodes per group was dissolved in isopropanol for quantitation. (Right) Proportions of certain bacteria determined by metagenome sequencing. One-way ANOVA with Tukey test was used for multiple comparisons. A *P* value of *<*0.05 was considered significant. Groups with different letters have significant differences. (B) Synchronized nematodes were cultured with different chemical reagents for 3 days and then stained with oil red O. Typical nematodes are shown in micrographs. After washing, oil red O from 50 nematodes per group was dissolved in isopropanol for quantitation. The OD at 510 nm was measured and three individual tests were performed for each assay. One-way ANOVA with Tukey test was used for multiple comparisons. A *P* value of *<*0.05 was considered significant. Groups with different letters have significant differences. (C) OP9 cells were differentiated by 1 μM rosiglitazone for 5 days. During this process, cells were treated with different metabolites. After differentiation, oil red O staining was performed. (D) RT-quantitative PCR was performed to determine the mRNA level of adipogenic differentiation-related genes in treated OP9 cells. In bar charts, data are shown as means ± SEM.

We next explored the role of gut microbes and intestinal metabolites in lipid accumulation. Caenorhabditis elegans was used as a model organism due to its short life cycle and ease in lipid droplet detection. Lactobacillus plantarum was low and unchanged among HF, HS, and control diets, Bifidobacterium longum was high and unchanged among the three diets, and Bifidobacterium animalis was reduced and Clostridium butyricum was increased in the HED groups (Fig. 5A). Compared to E. coli OP50, L. plantarum ZS2058 and B. animalis BB-12 feeding reduced whereas B. longum JCM7053 and C. butyricum MIYAIRI 588 enhanced lipid accumulation in C. elegans (Fig. 5A). Of note, E. coli was also reduced in HED-fed mice.

As changes of d-xylopyranose, 9,12-octadecadienoic acid, and stearic acid may have been mostly related to the reduction in bran and addition of lard in HED, we did not consider them microbe-derived metabolites. Levels of l-aspartic acid, cholestan-3-ol (5β, 3α), campesterol, and α-tocopherol were markedly changed between NC and HED groups, but the content of cholesterol was relatively stable (Fig. 4A). Compared with cholesterol, steroidal cholestan-3-ol (5β, 3α) and campesterol treatment significantly reduced lipid accumulation whereas α-tocopherol had no effect (Fig. 5B). We further confirmed these results using OP9 mammalian cells. Rosiglitazone-induced adipogenesis was inhibited by l-aspartic acid, cholestan-3-ol (5β, 3α), and campesterol treatment (Fig. 5C). Increased expression of *Fabp4*, *adiponectin*, *Plin1*, and *Cebpα* is indicative of adipocyte differentiation and lipid accumulation. In general, campesterol reduces the transcription of all four genes. l-Aspartic acid promotes the expression of *adiponectin* but shows an opposite effect on *Plin1* and *Cebpα*. Cholestan-3-ol (5β, 3α) can reduce *Cebpα* expression (Fig. 5D). Taken together, the results show that l-aspartic acid, cholestan-3-ol (5β, 3α), and campesterol may inhibit lipogenesis through different mechanisms.

### HED-modulated intestinal metabolites influence RAW 264.7 macrophage activation.

**FIG 6:**
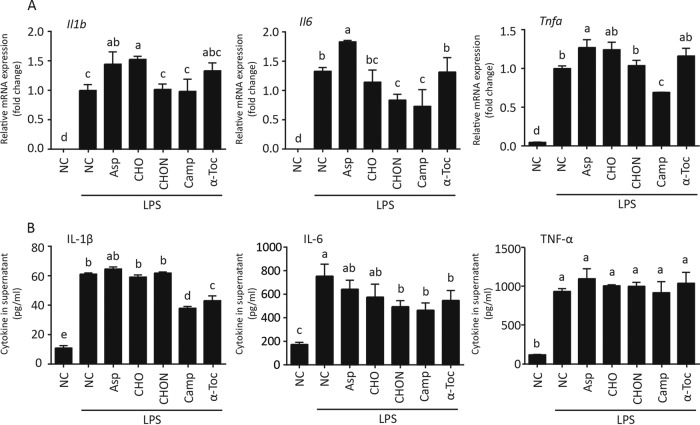
HED-modulated intestinal metabolites influence RAW 264.7 macrophage activation. RAW 264.7 macrophages were treated with 50 ng/ml of LPS combined with different metabolites for 24 h. Supernatant was collected for ELISA, and cells were then detached by trypsin. (A) RT-quantitative PCR was performed to measure the mRNA level of pre-*Il1b*, *Il6*, and *Tnfa* in RAW 264.7 macrophages. (B) Supernatant was used for ELISA to measure the content of mature IL-1β, IL-6, and TNF-α. One-way ANOVA with Tukey test was used for multiple comparisons. A *P* value of *<*0.05 was considered significant. Groups with different letters have significant differences. In bar charts, data are shown as means ± SEMs.

Macrophages, especially M1 polarized macrophages, are a critical mediator during obesity-associated chronic inflammation (32). We thus evaluated the roles of the above-mentioned metabolites in macrophage-associated inflammation. Effects of l-aspartic acid, cholesterol, cholestan-3-ol (5β, 3α), campesterol, and α-tocopherol on cytokine transcription and secretion were evaluated using lipopolysaccharide (LPS)-activated RAW 264.7 murine macrophages. l-Aspartic acid increased the mRNA but not mature protein of inflammatory interleukin 1β (IL-1β), IL-6, and tumor necrosis factor alpha (TNF-α). Cholestan-3-ol (5β, 3α) limited IL-6 production at both the mRNA and protein levels. Campesterol markedly reduced IL-6 and TNF-α transcription as well as IL-1β and IL-6 production (Fig. 6). These data suggest the potential anti-inflammatory role of cholestan-3-ol (5β, 3α) and campesterol as well as a possible proinflammatory role of l-aspartic acid.

## DISCUSSION

The development of type 2 diabetes (T2D) is not inevitable for those with prediabetes, and the incidence rate is approximately 25% over 3 to 5 years (25, 33). Prediabetes can be seen as a curable disease state and a checkpoint for T2D (34). Therefore, it is important to clarify pathophysiological changes associated with prediabetes. Prodiabetic gut microbiota induced by prodiabetic diets provide a mechanism of diabetes. In this study, we found that two different prodiabetic diets (HF and HS diets) did induce different gut microbiota but have similar gene profiles. *Lactobacillus* and *Bifidobacterium* were enriched by the HF diet and *Akkermansia* was enriched by the HS diet. It is reported that *Lactobacillus* and *Bifidobacterium* use both fat and sugar as nutrients, (35–37) while *Akkermansia* prefers sugar (38). This difference in physiological characteristic may be the cause of their differential enrichments. The similar gene profiles between HF- and HS-induced microbiota can be largely due to the fact that fat and sugar are both energy substances. After catabolism to currencies of energy such as acetyl-CoA, NADH, FADH_2_, and ATP, fat or sugar would share similar downstream energy utilization and anabolic processes under the regulation of similar gene sets.

*Lactobacillus*, *Bifidobacterium*, and *Akkermansia* were increased significantly in our prediabetic mice. These findings are in contrary to some previous studies showing their reduction in obese patients and their antiobesity effect (39–41). It is possible, however, that gut microbiota at the prediabetic state is different from those in diabetic patients and the elevation of *Lactobacillus*, *Bifidobacterium*, and *Akkermansia* represents a host’s early response to the changes in diet. In addition, it is worth noting that these changes are at the genus instead of species level, and changes in species are often different from that in the whole genus. de Goffau et al. found an increased abundance of the *Bacteroides* genus in children with β-cell autoimmunity. Meanwhile, Bacteroides fragilis was increased but B. thetaiotaomicron was decreased (42). Thus, species-specific adaptation to a certain gut environment describes the functional change of gut microbiota more accurately. In our study, although *Bifidobacterium* was enriched in HED groups, B. longum was unchanged and B. animalis was reduced in HED groups (Fig. 5A). Functionally, a strain of B. animalis reduced lipid accumulation in C. elegans, whereas B. longum enhanced this process (Fig. 5A). Therefore, reduced B. animalis can be a more credible biomarker of prediabetes than increased *Bifidobacterium*. Moreover, in the treatment of metabolic syndrome, supplementing B. animalis would be more beneficial than inhibiting the whole genus of *Bifidobacterium*.

Gut microbe-associated metabolites are critical mediators involved in the regulation of host metabolism by microflora. We studied weak polar metabolites and found a series of differences between HED and NC diet-derived metabolites, especially two sterols: campesterol and cholestan-3-ol (5β, 3α) (Fig. 4A). Campesterol, a plant-derived sterol, is also a substrate of microorganisms. Its negative correlation with metabolic syndrome has been reported (43). Cholestanols, which are cholesterol-derived metabolites, exist in serum and feces. Their reduction is also related to metabolic syndrome (43). In this study, we confirmed the inhibitory effect of campesterol and cholestan-3-ol (5β, 3α) on lipid accumulation *in vivo* (C. elegans) and *in vitro* (OP9 cells). Meanwhile, these metabolites can also limit macrophage inflammation (Fig. 6). Further mechanistic study of campesterol and cholestan-3-ol (5β, 3α) in suppressing metabolic syndrome is warranted.

## MATERIALS AND METHODS

### Diets and mice.

All procedures were approved by the ethics committee of Jiangnan University. Control chow diet (NC) diet and two types of high-energy diet (HED) were generated. The basis and additives are shown in Table 1; AIN-93M diet (44) was used as the basal diet. Male C57BL/6J (5 weeks age) mice were fed an NC diet, high-fat (HF) diet, or high-sucrose (HS) diet for 120 days. Free water and diet intake were stable during the experiment.

**TABLE 1:** Formulas for diets used in this study (1 kg)^*a*^

Component	Amt per diet		
	NC	HF	HS
AIN-93M basal diet (g)	800	520	520
Lard (g)	0	261	131
Sucrose (g)	0	49	349
Bran (g)	200	170	0
Energy density (kcal/100 g)	286.4	440.17	440.17

a Lard and sucrose provided excess energy. Wheat bran was used as a filler to ensure the same energy density between two high-energy diets. The energy of lard per 100 g is considered 902 kcal, and the energy of sucrose per 100 g is considered 387 kcal. These data are according to National Nutrient Database for Standard Reference Legacy Release, United States Department of Agriculture Agricultural Research Service (https://ndb.nal.usda.gov/ndb/search/list?home=true).

### Blood glucose measurement.

After 10 h of fasting, the basal glucose level was detected using a glucometer (Roche). Mice were orally injected with 1.5 g of glucose per kg of body weight (40% glucose solution), and blood glucose levels were recorded. These measurements were obtained in a blinded manner.

### Next-generation sequencing and bioinformatic analysis for diversity analysis.

Total genome DNA from stool samples was extracted using a PowerSoil DNA isolation kit (12888-100; Qiagen, Shanghai, China) according to the manufacturer’s instructions. The samples for sequencing 16S rRNA gene V4 regions were prepared following the protocol of 16S metagenomic sequencing library preparation of Illumina. To generate amplicons, the V4 region of 16S rRNA was amplified using specific primers (forward primer, 5′-AYTGGGYDTAAAGNG-3′; reverse primer, 5′-TACNVGGGTATCTAATCC-3′) with a 28-cycle PCR. All PCRs were carried out with KAPA HiFi HotStart (KR0370; KAPA Biosystems, MA). The PCR products were then purified with magnetic beads (Agencourt AMPure XP; Beckman, CA). The second round of PCR amplification was implemented to introduce dual indices and sequencing adapters. Similar PCR conditions were used except that the cycle number was decreased to eight. AMPure XP beads were used to clean up the final indexed product. The purified products were quantified using a fluorometric quantification method and pooled into a library after normalization. The DNA sequencing was performed on an Illumina Hiseq 2500 to generate pair-end 250-bp reads. In this study, the number of reads = 128,869 ± 2,236 (mean±standard error of the mean [SEM]). The data analysis was performed by QIIME 1 platform (45). Raw sequencing data were filtered using FASTQC according to the phred scores, and the reads were trimmed if the average phred score in the window (5 bp in size; 1-bp step length) was less than 20 (46). Reads containing ambiguous ‘N’ or with lengths of <150 bp were discarded. Paired reads were merged into a tag sequence according to their overlap. Chimera reads and the corresponding operational taxonomic units (OTUs) were removed by ChimeraSlayer (47) and QIIME scripts. High-quality sequences without chimeras were clustered into OTUs using Uclust with a similarity of 97% (48).

We chose 0.001% as the threshold for filtering low-abundance OTUs; i.e., only OTUs with read counts of >0.001% of the total reads of all samples were kept. The longest sequence of each OTU was selected as a representative sequence which was annotated by comparison to the Greengenes database (release 13.5; http://greengenes.secondgenome.com/) by the RDP-classifier method (49, 50).

Microbial diversity was measured by a series of OTU-based analyses of alpha- and beta-diversity implemented in the QIIME pipeline. Alpha-diversity and beta-diversity analyses were performed based on OTUs normalized by a standard of sequence number corresponding to the sample with the least sequences. Indices, including observed-species, Chao1, Shannon, Simpson, and good-coverage indices, were calculated and displayed with R program through rarefactions to indicate alpha-diversity, the diversity of species in a sample. Beta-diversity was used to evaluate differences of samples in species diversity and was characterized by both weighted and unweighted UniFrac methods. Other indices, including Bray-Curtis and Pearson, were also used to indicate beta-diversity. Subsequently, principal-coordinate analysis (PCoA) based on Bray-Curtis distance or Pearson distance was performed with iterative algorithm. Hierarchical clustering analysis was performed based on unweighted UniFrac and weighted UniFrac conducted by QIIME. The Vegan 2.0 package was used to generate a PCoA figure. Analysis of similarity (ANOSIM) was used to test the significance among groups. An online LEfSe analysis was adopted to search for biomarkers of different groups (http://huttenhower.sph.harvard.edu/galaxy) (51). According to the LEfSe analysis, species with *P* values of <0.05 in Kruskal-Wallis (KW) sum-rank test and LDA score of >4.0 were plotted.

### Next-generation sequencing and bioinformatic analysis for metagenomic analysis.

Genomic DNA was sonicated to a 100- to 800-bp size range. Then libraries were constructed using an NEBNext DNA kit (E6040; New England BioLabs, Beijing, China) according to the instructions. DNA fragments (>200 bp) were PCR amplified with Illumina adapter-specific primers. Libraries whose average insert size was about 350 bp were sequenced with a HiSeq X Ten sequencer (Illumina, CA) using the paired-end method.

Illumina raw reads were filtered with the following constraints: (i) reads with more than 2 ambiguous N bases were removed, (ii) reads with less than 80% high-quality bases (phred score ≥ 20) were removed, and (iii) 3′ ends of reads were trimmed to the first high-quality base. Then filtered metagenomic reads were assembled by Megahit (version 1.0.5) (52) into contigs in a time- and cost-efficient way, with the following parameters: –min-contig-len = 150, –k-min = 27, –k-max = 123, –k-step = 8, and –min-count = 1. All assembled contigs were submitted to MetaProdigal (version 2.6.3) (53) for gene calling using the default parameters. We aligned all reads to genes with Bowtie2 and calculated the gene coverage using bedtools (version 2.26) (54).

We mapped the predicted genes to NCBI bacteria, archaebacterial, and virus nonredundant genome databases with Diamond (55). The alignment result was then submitted to Megan (version 6) to estimate the taxonomic and functional compositions with weighted LCA algorithm (56). The taxonomic analysis was performed with NCBI bacterial, archaeal, and viral nonredundant genome databases. The functional analysis was conducted by mapping genes to Kyoto Encyclopedia of Genes and Genomes (KEGG) (57) and SEED (58). Enrichment of gene sets was calculated via an online tool GSEA (http://www.gsea-msigdb.org/gsea/index.jsp).

The Vegan 2.0 package was used to generate a PCA and heat map figures based on taxonomy or gene matrix. Analysis of variance (ANOVA) was used to test the significance among groups. Benjamini FDR was used for correction for multiple tests (*P < *0.05).

### Microbes and mammalian cells.

Escherichia coli strain OP50 was preserved by this lab. Lactobacillus plantarum ZS2058, Bifidobacterium animalis subsp. *lactis* BB-12, and Bifidobacterium longum subsp. *longum* (CGMCC 1.3048, JCM 7053) were kindly gifted by Haiqin Chen, School of Food Science and Technology, Jiangnan University. Clostridium butyricum strain MIYARI 588 was cultured from the commercially available MIYAIRI 588 (CBM 588) Miyarisan tablets. E. coli strain OP50 was maintained in LB media at 37°C in a normoxic incubator. L. plantarum ZS2058, B. animalis *subsp. lactis* BB-12, B. longum subsp. *longum*, and *C. butyricum* strain MIYARI 588 were cultured in MRS medium (containing 0.5% [wt/vol] cysteine) at 37°C in an anaerobic incubator. Bacterium number was counted through a microscope.

OP9 embryonic stem cells and RAW 264.7 macrophages were provided by the Chinese Academy of Sciences Shanghai Institutes for Biological Sciences, China. OP9 cells were maintained in alpha minimum essential medium supplemented (α-MEM) with 10% (vol/vol) fetal bovine serum (FBS; 10099141; Gibco, MA) and differentiated by rosiglitazone in α-MEM (32561102; Gibco, MA) supplemented with 20% (vol/vol) newborn calf serum (NBCS; 16010159; Gibco, MA) for 5 days. RAW 264.7 cells were maintained in Dulbecco’s modification of Eagle’s medium (DMEM; 11995-065; Gibco, MA) supplemented with 5% (vol/vol) FBS and activated by 50 ng/ml of LPS (L4516; Sigma, MO) for 24 h.

### GC-MS analysis of weak polar metabolites.

Stool samples were homogenized in methanol-water (5/1) to quench and ultrasonicated to release metabolites. Then a double volume of chloroform was added. After vortexing and centrifugation, the organic phase was collected and evaporated to dryness, followed by silylation derivatization for gas chromatography-mass spectrometry (GC-MS). Samples were analyzed on a GC-MS detector (TSQ 8000 evo; Thermo Scientific) with an RTX-5MS column (30 m by 0.25 mm; 0.25-μm film thickness; Restek, Bellefonte, PA). Conditions of GC-MS were the same as in a previous study (59). Peak area was recorded for relative quantification.

### Chemical reagents.

l-Aspartic acid (A9256), cholesterol (C8667), cholestan-3-ol (5β, 3α) (C2882), campesterol (C5157), and α-tocopherol (258024) used as standards and stimuli were purchased from Sigma-Aldrich, MO. l-Aspartic acid was dissolved in deionized water and the other reagents were dissolved in ethanol. For cultured cells, the final concentrations of these five reagents were 3 μM, 1 μM, 1 μM, 1 μM, and 1 μM. For nematodes, the final concentrations of these five reagents were 30 μM, 10 μM, 10 μM, 10 μM, and 10 μM.

### Nematodes.

Wild-type Caenorhabditis elegans nematodes were kindly gifted by Zhennan Gu, School of Food Science and Technology, Jiangnan University. The general culture method is described in WormBook (http://www.wormbook.org). In brief, C. elegans was maintained in nematode growth medium (NGM) plates at 22°C, and E. coli strain OP50 was added as the diet. To research the function of microbes and metabolites, synchronous cultures of C. elegans were used. For microbes, 1 × 10^6^/ml of target bacterium or E. coli strain OP50 was added into S basal medium and then worms were transferred in. For metabolites, the metabolite and E. coli strain OP50 were added into S basal medium and then worms were transferred in. C. elegans grew in liquid medium on a shaker at 22°C. After 48 h of treatment, C. elegans was collected via free dropping and washed with M9 buffer.

### Oil red O staining.

For cultured mammalian cells, an oil red O stain kit (ab150678; Abcam, Cambridge, UK) was used and manufacturer instructions were followed. For C. elegans, worms were washed with 1× phosphate-buffered saline (PBS) twice and settled by gravity. After 15 min of 4% paraformaldehyde fixation, another wash was performed. Then 60% isopropanol was added and worms were stained in filtered oil red O staining solution (60% oil red O stock solution [5 mg/ml of isopropanol] and 40% water) overnight at room temperature. Worms were then washed with PBS and observed through an inverted microscope (Eclipse; Nikon). For quantification, oil red O was dissolved by isopropanol and then the optical density (OD) at 510 nm was measured. For an individual assay, 50 nematodes from each group were dissolved. Three individual tests were performed for each assay.

### RNA extraction and quantitative PCR.

Total RNA was extracted from the cultured cells using TRIzol reagent (15596026; Thermo Fisher). cDNA was synthesized using the PrimeScript reverse transcription (RT) reagent kit with gDNA Eraser (RR047A; TaKaRa, Beijing, China). Real-time PCR analysis using Power SYBR green PCR master mix (4367659; Thermo Fisher) was carried out on a CFX 96 real-time PCR system (Bio-Rad). The primer sequences used are as follows (5′ to 3′): *Fabp4*, AAGGTGAAGAGCATCATAACCCT (forward) and TCACGCCTTTCATAACACATTCC (reverse); *Adiponectin*, TGTTCCTCTTAATCCTGCCCA (forward) and CCAACCTGCACAAGTTCCCTT (reverse); *Plin1*, GGGACCTGTGAGTGCTTCC (forward) and GTATTGAAGAGCCGGGATCTTTT (reverse); *Cebpa*, CAAGAACAGCAACGAGTACCG (forward) and GTCACTGGTCAACTCCAGCAC (reverse); *Il1b*, GCAACTGTTCCTGAACTCAACT (forward) and ATCTTTTGGGGTCCGTCAACT (reverse); *Il6*, TAGTCCTTCCTACCCCAATTTCC (forward) and TTGGTCCTTAGCCACTCCTTC (reverse); and *Tnfa*, CCCTCACACTCAGATCATCTTCT (forward) and GCTACGACGTGGGCTACAG (reverse). Relative mRNA expression was calculated following the formula: fold = 2^−△△^*^CT^*, where *CT* is threshold cycle.

### ELISA.

The supernatant of cultured cells was collected and cell debris was removed through centrifugation at 12,000 × *g* and 4°C. Then cytokines in the supernatant were detected via a double-sandwich method-based enzyme-linked immunosorbent assay (ELISA). Commercially available murine IL-1β (900-K47), murine IL-6 (900-K50), and murine TNF-α (900-K54) ELISA kits (Peprotech, Beijing, China) were adopted in this study. All operations were performed according to the instructions.

### Statistics.

Except for next-generation sequencing-associated statistical analysis, two-tailed Student’s *t* test was used for the statistical comparison of two groups and one-way ANOVA was used for multiple comparisons. In the case that all groups shared identical sample sizes the Tukey test was adopted; otherwise the Bonferroni test was adopted. A *P* value of <0.05 was considered significant.

### Data availability.

Raw sequencing data have been uploaded to the Sequence Read Archive. The accession number is PRJNA565559. The metabolomics data have been uploaded to BioStudies. The accession number is S-BSST281 (https://www.ebi.ac.uk/biostudies/studies/S-BSST281).
