# Supplementary figures and images for: Distinct Gut Microbiota Induced by Different Fat-to-Sugar-Ratio High-Energy Diets Share Similar Pro-obesity Genetic and Metabolite Profiles in Prediabetic Mice
Source: mSystems. 2019 Oct 8;4(5):e00219-19. doi: 10.1128/mSystems.00219-19 (PMC6787563; doi:10.1128/mSystems.00219-19)

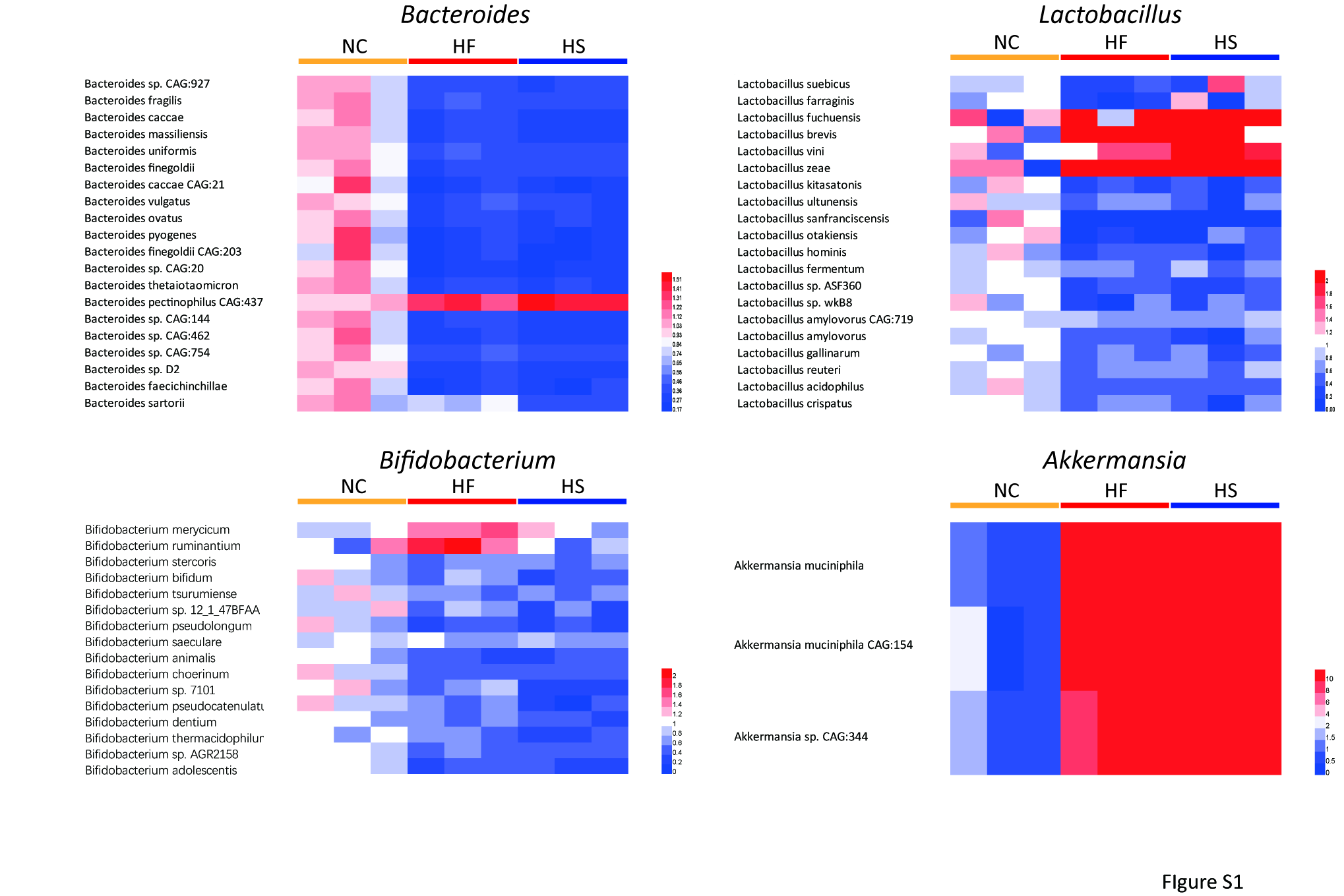

Supplement: FIG S1 [file mSystems.00219-19-sf001.tif]

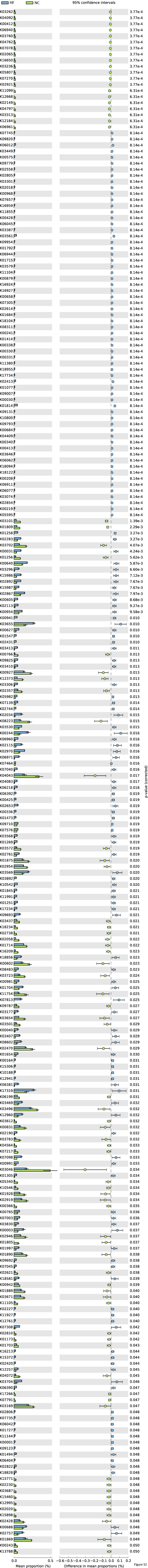

Supplement: FIG S2 [file mSystems.00219-19-sf002.tif]

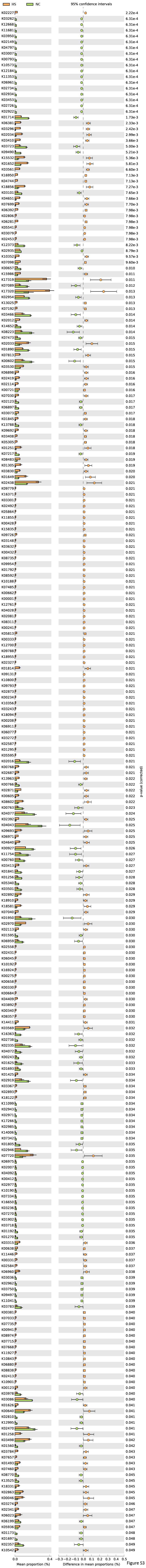

Supplement: FIG S3 [file mSystems.00219-19-sf003.tif]

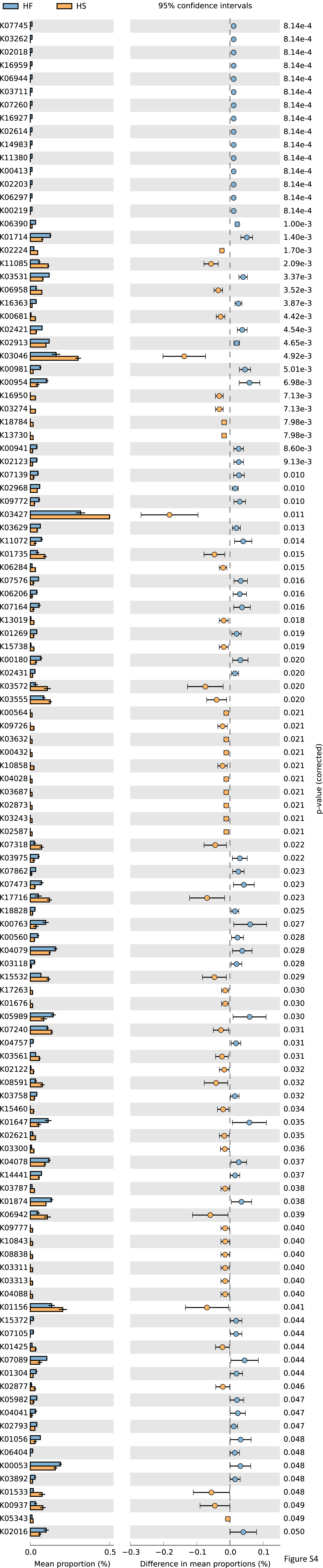

Supplement: FIG S4 [file mSystems.00219-19-sf004.tif]

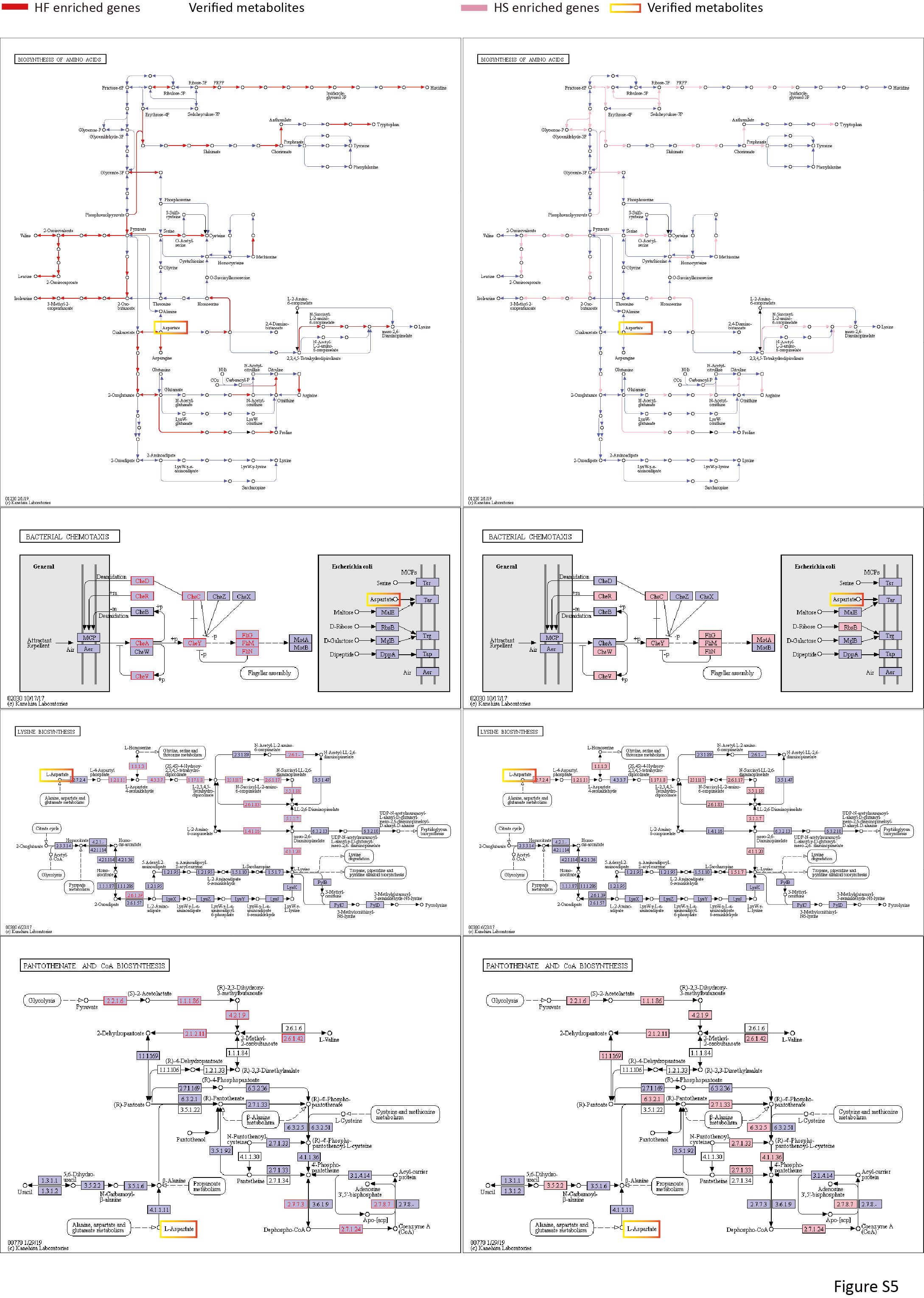

Supplement: FIG S5 [file mSystems.00219-19-sf005.tif]

■ HF enriched genes □

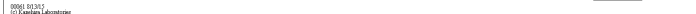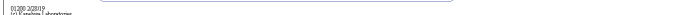

■ HS enriched genes

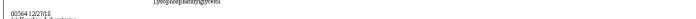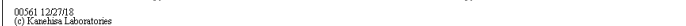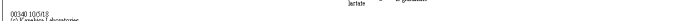

Figure S6

Supplement: FIG S6 [file mSystems.00219-19-sf006.pdf]
